# Role of Heparan Sulfate 2-O-Sulfotransferase in Prostate Cancer Cell Proliferation, Invasion, and Growth Factor Signaling

**DOI:** 10.1155/2011/893208

**Published:** 2011-10-23

**Authors:** Brent W. Ferguson, Sumana Datta

**Affiliations:** Department of Biochemistry/Biophysics, Texas A&M University, MS 2128, College Station, TX 77845, USA

## Abstract

Heparan-sulfate proteoglycans (HSPGs) are required for maximal growth factor signaling in prostate cancer progression. The degree of sulfate modification on the covalently attached heparan sulfate (HS) chains is one of the determining factors of growth factor-HSPG interactions. Sulfate groups are transferred to HS chains via a series of O-sulfotransferases. In the present study, we demonstrate that Heparan sulfate 2-O-sulfotransferase (2OST) is essential for maximal proliferation and invasion of prostate cancer cells in the LNCaP-C4-2B model. We also show that a decrease in invasion due to 2OST siRNA is associated with an increase in actin and E-cadherin accumulation at the cell surface. 2OST expression correlates with increasing metastatic potential in this model. We demonstrate that 2OST expression is upregulated by the stress-inducible transcription factors HIF1*α*, ATF2, and NF*κ*B. Chromatin immunoprecipitation analysis suggests that HIF1*α* and ATF2 act directly on the 2OST promoter, while NF*κ*B acts indirectly.

## 1. Introduction

Heparan sulfate proteoglycans (HSPGs) are ubiquitous cell surface molecules that consist of a protein core with attached heparan sulfate (HS) glycosaminoglycan chains. HSPGs are extremely important in both development and cancer progression due to their regulation of cellular processes such as angiogenesis, tumor growth, proliferation, tumor invasion and metastasis. HSPGs control various processes by modulating a variety of growth factor signaling pathways such as Sonic Hedgehog (SHH), FGF, VEGF, and TGF*β* [1–3]. These signaling pathways are abnormally activated in many cancers including prostate cancer [4, 5].

HSPGs such as Syndecan-1 and Perlecan (Pln) are involved in the regulation of tumor growth and proliferation of prostate cancer cells [6–9]. Our group, along with collaborators, demonstrated the association of high levels of Pln protein with 54% of advanced prostate cancer tumors and its role in tumor cell proliferation by regulating SHH signaling [8]. This led to the hypothesis that a subset of prostate cancers reach advanced stage by increasing growth factor signaling through increasing the amount of Pln coreceptor in the extracellular matrix. However, the other 46% of advanced prostate cancers showed no increase in Pln protein levels compared to more benign tissue, suggesting another mechanism is in play. Interestingly, in the LNCaP-C4-2B cell line series, a well-known model of prostate cancer progression [10–12], we have shown that SHH signaling increases with increasing metastatic potential but Pln protein levels do not [8]. Instead, in this cell line series, Pln isolated from more highly metastatic cell lines binds more SHH than an equal amount of Pln from more benign cell lines. This data suggested an alternative mechanism, whereby during prostate cancer progression, cells produce a different, more efficient isoform of Pln protein to increase SHH signaling rather than simply expressing more of the same isoform as before. Given the bipartite structure of HSPGs and the known contribution of their sugar chains to the regulation of growth factor signaling, differential structure of the sugar chains is an obvious possibility in the generation of different Pln isoforms.

The ability of HS to bind growth factors such as FGF, VEGF, and hepatocyte growth factor has been shown to largely depend on the amount of HS sulfation [13–16]. The general rule is that the higher degree of sulfation on the HS chain the greater the binding to growth factors. One possible way of increasing the amount of sulfation on HS chains is for the cell to increase the expression of the different O-sulfotransferases (OSTs) that act upon the glycosaminoglycan chain [17]. We have chosen to investigate the impact of cellular stress, a common characteristic of tumor progression, on the expression of these enzymes and the effect of changes in enzyme expression on cancer cell behavior. 

Solid tumors, such as prostate cancer, make up approximately 90% of all cancers and result in significant mortality due to cell invasion and metastasis to distant vital organs such as the brain and lungs [18]. The rapid proliferation associated with formation of a solid tumor induces stress in the tumor, such as hypoxia. Cells respond to hypoxic stress by stabilizing the transcription factor hypoxia-inducible factor 1*α* (HIF1*α*) which heterodimerizes with HIF1*β* and activates transcription of its target genes [19]. HIF1*α* is also stabilized in response to metabolic stress produced by mitochondrial mutations that increase production of reactive oxygen species (ROS) [20]. Finally, HIF1*α* activity is increased in response to androgen signaling in androgen-sensitive prostate cancer cells [21]. The HIF1 heterodimer binds to specific sites called hypoxia response elements (HREs) within the promoter of a target gene. HIF1*α* is a logical candidate to control OST expression, because it is overexpressed in prostate tumors [22] as well as in human prostate cancer cell lines [23]. The accumulation of reactive oxygen species (ROS) is another source of cellular stress shown to be associated with solid tumors [24]. ROS activates a number of stress-activated protein kinases. These kinases activate transcription factors that stimulate the expression of genes involved in cancer progression. We have focused on two ROS-inducible transcription factors, NF*κ*B and ATF2, as well as the protein kinase p38 MAPK. 

We queried the Oncomine database and found that 2OST expression is upregulated in prostate carcinoma compared to normal tissue in three previously published reports (Varambally, Luo, and Liu). This evidence led us to further investigate the role of 2OST in prostate cancer and its progression. We have found evidence that the level of expression of the 2OST enzyme correlates with metastatic potential in the LNCaP-C4-2B cell line model. We also demonstrate that the proliferation of these cells as well as their migration is dramatically reduced when 2OST expression is knocked down. Decreased migration of C4-2B cells through matrigel as a result of 2OST knockdown correlates with increases in cell adhesion via E-cadherin accumulation. We also demonstrate that 2OST is required for optimal FGF, TGF*β*, and SHH signaling in C4-2B. Complex formation between the SHH and the HSPG Pln, an interaction known to be required for optimal signaling, is also decreased upon inhibition of 2OST expression. Finally, we show that maximal 2OST expression requires the stress-inducible transcription factors HIF1*α*, ATF2, and NF*κ*B. Chromatin immunoprecipitation studies suggest that HIF1*α* and ATF2 act directly on the 2OST promoter while NF*κ*B acts indirectly. Taken together, our studies strongly suggest that increases in 2OST levels, and presumably modification of HS chains on heparan sulfate proteoglycans, account for the increased metastatic behavior of a well-characterized model of prostate cancer progression. Furthermore, these studies are consistent with the possibility that the 46% of advanced human prostate cancer tumors that failed to show an increase in Perlecan protein levels over normal tissue may have progressed to a metastatic state through production of a more efficient form of highly sulfated Perlecan due to upregulation of 2OST expression.

## 2. Materials and Methods

### 2.1. Cell Lines and Culture Conditions

The LNCaP series (LNCaP, C4, C4-2, C4-2B) were obtained from Dr. L. Chung and grown at 37°C and 5% CO_2_ in T-medium supplemented with 5% fetal bovine serum (Invitrogen).

### 2.2. Reagents and Antibodies

Primary antibodies purchased from Santa Cruz Biotechnology (SCBT) are as follows: anti-HS2ST1 (Santa Cruz no. sc-130779), anti-*β*-catenin (sc-7963, 1 : 500), anti-phospho-ERK (sc-81492, 1 : 1000), anti-phospho-SMAD2/3 (sc-11769, 1 : 1000), anti-phospho-ATF2 (sc-52941, 1 : 500), and anti-SHH (sc-9024, 1 : 500). Anti-E-cadherin was purchased from Invitrogen (no.18-0223). Anti-Pln antibody was purchased from US Biological (no. H1890, 1 : 1000) and anti-HIF1*α* antibody was purchased from Novus (NB100, 1 : 500). Purified anti-*β*-actin antibody (A5316, 1 : 1000) was purchased from Sigma Aldrich. Anti-mouse HRP and antirabbit HRP secondary antibodies (1 : 10,000) were purchased from Jackson Labs. Antibodies for chromatin immunoprecipitation were used at a final concentration of 0.3 mg/mL and as follows: anti-HIF1*α* (Novus Biologicals no. NB100), anti-ATF2 (SCBT sc-6233), and anti-NF*κ*B p65 (SCBT sc-109). SB202190 (Cat. no. S7067) was purchased from Sigma Aldrich.

### 2.3. Western Blotting and Coimmunoprecipitations

Isolation of total protein was done using the Mammalian Cell Lysis Kit from Sigma Aldrich (no. MCL-1KT) according to manufacturer's protocol. Phosphatase inhibitors were used for each sample (Sigma Aldrich no. P0044). Samples were prepared and run on 15% SDS-PAGE and transferred onto nitrocellulose. Western blots were developed with Pierce ECL Western Blotting Substrate (no. 32106). Images and densitometry were obtained on a BioRad ChemiDoc XRS machine using Quantity One software. Densitometry values represent the mean of two independent experiments. Pln-SHH coimmunoprecipitations were performed as previously described [8]. Briefly, conditioned medium from C4-2B cells treated with either control siRNA or 2OST siRNA was collected when cells reached 80%–90% confluence in 100 × 15 culture dishes. Equivalent amounts of conditioned medium were immunoprecipitated with anti-Pln antibody and the bound complexes were run on SDS-PAGE. Bound SHH was observed by immunoblotting.

### 2.4. Transient Transfections

Transient transfections of siRNA were performed using Lipofectamine 2000 reagent (Invitrogen no. 11668027) for siRNA directed towards HIF1*α*, NF*κ*B, or ATF2 (Ambion) according to manufacturer's protocol. 2OST siRNA (Sigma no. SASI_Hs01_00214049 and SASI_Hs01_00214052) was performed using Oligofectamine transfection reagent (Invitrogen no. 12252011) according to manufacturer's protocol. Briefly cells were cultured in 6-well plates at allowed to attach for 24 hours. siRNA was applied and cells were harvested for either protein or RNA after 24 hours. Scrambled siRNA was used as the negative control. Transient transfection of stabilized HIF1*α* (stHIF1*α*) (a kind gift from Dr. Eric Huang) as well as p2OST-lacZ reporter plasmid were performed using Lipofectamine 2000 reagent according to manufacturer's protocol.

### 2.5. RNA Isolation and Real-Time PCR

Cells were grown to 80%–90% confluence, scraped, centrifuged and washed with PBS. RNA isolation was performed with Qiagen RNEasy Mini-kit (no. 74104) according to manufacturer's protocol. 2 *μ*g of RNA was used in each DNAse I reaction using DNAse I Amplification Grade from Invitrogen (no. 18068). Reverse transcription was performed with oligo dT and random hexamer primers using SuperScript III reverse transcriptase from Invitrogen (no. 18080044). Real-time PCR was performed using Taqman Gene Expression Assays with Taqman Universal PCR Master Mix from Applied Biosystems (no. 4324018). Each sample from two independent experiments was run in triplicate at three different concentrations and normalized to levels of 18S rRNA. Fold increase/decrease comparisons were calculated using the delta-delta CT method. Data for each sample is presented as the mean fold change compared to control and error is presented as standard deviation. Reactions were performed using a BioRad C1000 Thermal Cycler machine.

### 2.6. Proliferation Assay

BrdU (Sigma-Aldrich) was added to the cells at a final concentration of 20 *μ*M and allowed to incubate for two hours. Immunocytochemistry on cell lines with scrambled or 2OST siRNA was carried out with anti-BrdU (Becton Dickinson) and HRP-conjugated secondary antibodies (Boehringer Mannheim) using standard techniques. Proliferation was quantified by counting the number of BrdU-positive cells in a field of 100 done in triplicate.

### 2.7. Migration Assay

Cell migration assays were performed using Matrigel Invasion Chambers from BD Biocoat (no. 354480) and control inserts (no. 354578) according to manufacturer's protocol. Number of C4-2B cells migrated through Matrigel was counted in control cells (scrambled siRNA treated) and experimental cells (2OST siRNA treated) in four separate fields in three independent experiments. The same experiment was performed in control inserts. The average number of invading cells through Matrigel (*n* = 12) was normalized to the average number of cells on control inserts (*n* = 12) to determine percent invasion. Error bars indicate standard deviation.

### 2.8. Phalloidin Staining and E-cadherin-Phalloidin Double Labeling

C4-2B cells were treated with scrambled siRNA or 2OST siRNA as described above and cultured on glass coverslips coated in poly-L-lysine (BD Biocoat no. 354085). Cells were washed with PBS, fixed in 3% formaldehyde, permeabilized with PBST, treated with 1 : 1000 dilution of FITC-phalloidin (Dr. B. Perkins), and mounted in VectaShield mounting medium with DAPI (Vector Laboratories). Cells were imaged with fluorescence microscopy. Individual cells were first chosen by their nuclei under the DAPI channel, then the number of actin foci per cell were counted for each treatment (*n* = 15 per treatment). The double labeling experiment was performed in much the same way as the phalloidin staining of C4-2B cells treated with either control or 2OST siRNA with a few modifications. Following permeabilization with PBST fixed cells were blocked with 0.1% FBS in PBS. *α*-E-cadherin antibody was added to a final concentration of 0.5 *μ*g/mL for 30 minutes then cells were washed with PBS three times. A 1 : 1000 dilution of *α*-mouse Alexa 488 secondary antibody was added for 30 minutes then followed with three more rounds of washing. Cells were then treated with a 1 : 40 dilution of Alexa 546 phalloidin for 20 minutes then washed again. Cells were air dried and mounted on microscope slides in Vectashield mounting medium with DAPI.

### 2.9. p2OST Reporter Plasmid and *β*-galactosidase Assays

Two different constructs from the 2OST promoter were amplified by PCR and cloned into the pBLUE-TOPO TA vector (Invitrogen no. K4831-01). “Full-length” p2OST consists of 2500 bases upstream and 435 bases downstream of the transcription start site while “4C” p2OST consists of 1500 bases upstream and 435 bases downstream of the transcription start site. Primers used to amplify the “full-length” promoter were 5′-tcaaacggtgaaccaagacgctgt-3′ and 5′-gaaacccgctgctcggg-3′. Primers used to amplify the “4C” promoter were: 5′-actccggtgtagtcccttaaca-3′ and 5′-gaaacccgctgctcggg-3′. *β*-galactosidase assays to evaluate the amount of transcription from each of the p2OST constructs were performed using the *β*-gal Assay Kit (Invitrogen no. K1455-01) according to manufacturer's protocol.

### 2.10. Chromatin Immunoprecipitation

C4-2B cells were cross-linked by adding formaldehyde directly to cell culture medium to a final concentration of 1%. Cross-linking was allowed to proceed for 10 min at room temperature then stopped with addition of glycine to a final concentration of 0.125 M. Cells were washed twice with ice-cold PBS and swollen with PBS for 10 minutes at 37°C. Cells were scraped, washed once with PBS then pelleted by centrifugation. Pellets were resuspended in Cell Lysis Buffer (5 mM PIPES pH 8.0, 85 mM KCl, 0.5% Triton X-100, protease inhibitor cocktail) for 10 minutes on ice. Cellular extract was pelleted by centrifugation then nuclei were resuspended in Nuclei Lysis Buffer (50 mM Tris-Cl pH 8.0, 10 mM EDTA, 1% SDS, protease inhibitor cocktail) for 10 minutes on ice. Total chromatin was then sonicated for twelve 20-second pulses at setting 2. After centrifugation at 12,000 g chromatin was precleared with Protein A/G Plus Beads then divided into aliquots. Antibody was added to each aliquot for a final concentration of 0.3 mg/mL and incubated on a rotating platform overnight at 4°C. Antibody-protein complexes were immunoprecipitated with Protein A/G Plus Beads. Samples were washed extensively and eluted in Elution Buffer (50 mM NaHCO_3_, 1% SDS). Bound DNA fragments were isolated and analyzed by PCR. Primers used to amplify the region of the 2OST promoter from −1157 to −707 (H1) were 5′-ttaaaagcacaaatcgcactca-3′ and 5′-gaaaagggtggggaggact-3′. Primers used to amplify the region from −581 to −231 (H2 and A2) were 5′-ggcaccagacacccactc-3′ and 5′-aagaaggcggggctaaaac-3′. Primers used to amplify the region from −1499 to −1219 (A1) were 5′-actccggtgtagtcccttaaca-3′ and 5′-tttttaaatgatgttcgttgtcttc-3′. Primers used to amplify the region from +224 to +435 (N1) were 5′-gactggagaggcgagaagg-3′ and 5′-gaaacccgctgctcggg-3′. Primers used to amplify the region from −176 to +53 (N2) were: 5′-caaccgtaaaccgaaccaag-3′ and 5′-tccctctcttccttccttcc-3′.

### 2.11. Promoter Analysis

Prediction of transcription factor binding sites in the human 2OST promoter was done with the ALGGEN-PROMO prediction program.

## 3. Results

### 3.1. OST Expression is Upregulated in Prostate Carcinoma

To investigate the role of 2OST in prostate cancer, we explored the Oncomine database (https://www.oncomine.org/). The Oncomine concept that was chosen was “Prostate Carcinoma versus Normal—Top 10% overexpressed.” Within this concept we found three different studies that included 2OST in the cDNA microarray. Each of these studies show that 2OST expression is significantly upregulated in prostate carcinoma compared to normal tissue (Table 1). This evidence led us to further examine the role of 2OST in prostate cancer using the LNCaP-C4-2B model of prostate cancer progression.

### 3.2. 2OST Expression Correlates with Metastatic Potential

The LNCaP-C4-2B cell line model of prostate cancer progression was originally identified in the laboratory of Dr. Leland Chung [11, 12]. This series of lines was established via serial transplantation of cancer cells into nude mice. The LNCaP cell line was originally derived from a supraclavicular lymph-node metastasis of a primary prostatic carcinoma [25]. LNCaP cells mimic many of the characteristics of early stage prostate cancer in that they are weakly tumorigenic when inoculated into nude mice, their growth is androgen-sensitive and they secrete low levels of PSA [25]. The C4 subline shows higher levels of PSA expression than LNCaP, produces approximately 10 times more colonies in soft agar and are still androgen-sensitive in their growth. The next subline in the series, C4-2, is highly tumorigenic on its own, displays androgen insensitive growth and metastasizes to both the lymph node and bone. The final subline of the series, C4-2B, mimics the most advanced stage of prostate cancer. It is androgen-insensitive, secretes the highest levels of PSA, and rapidly metastasizes to bone. 

**Figure 1:**
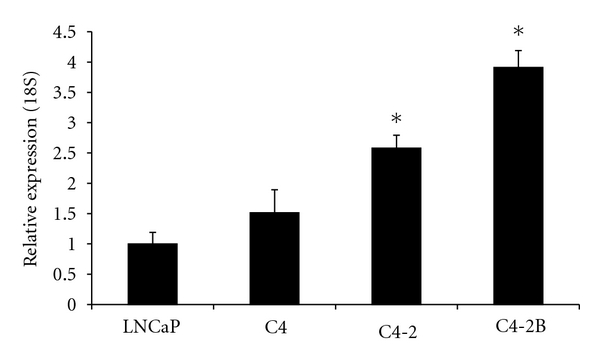
2OST expression correlates with metastatic potential. A 2OST mRNA levels in LNCaP series of prostate cancer progression. 2OST mRNA was assayed by qRT-PCR and normalized to 18S rRNA. All samples were run in triplicate and overall 2OST message levels compared by setting 2OST levels in LNCaP to 1. Samples are presented by increasing metastatic potential (LNCaP, C4, C4-2, C4-2B). Error bars indicate standard deviation. Asterisk indicates *P* < 0.05.

To ask if 2OST might play a role in the invasive and metastatic potential of cell lines in the LNCaP-C4-2B series we first evaluated basal expression of the 2OST gene in each of the cell lines. Quantitative real-time PCR analysis of 2OST mRNA levels using the weakly tumorigenic LNCaP line as a normalization standard reveals a step-wise increase in 2OST expression as the cell lines increase in their metastatic potential (Figure 1). Levels of 2OST increased fourfold in the C4-2B cell line as compared to LNCaP. This result demonstrates a direct correlation between increasing characteristics of advanced prostate cancer cells and 2OST expression.

### 3.3. Knockdown of 2OST Expression Results in Decreased Prostate Cancer Cell Proliferation and Migration

**Figure 2:**
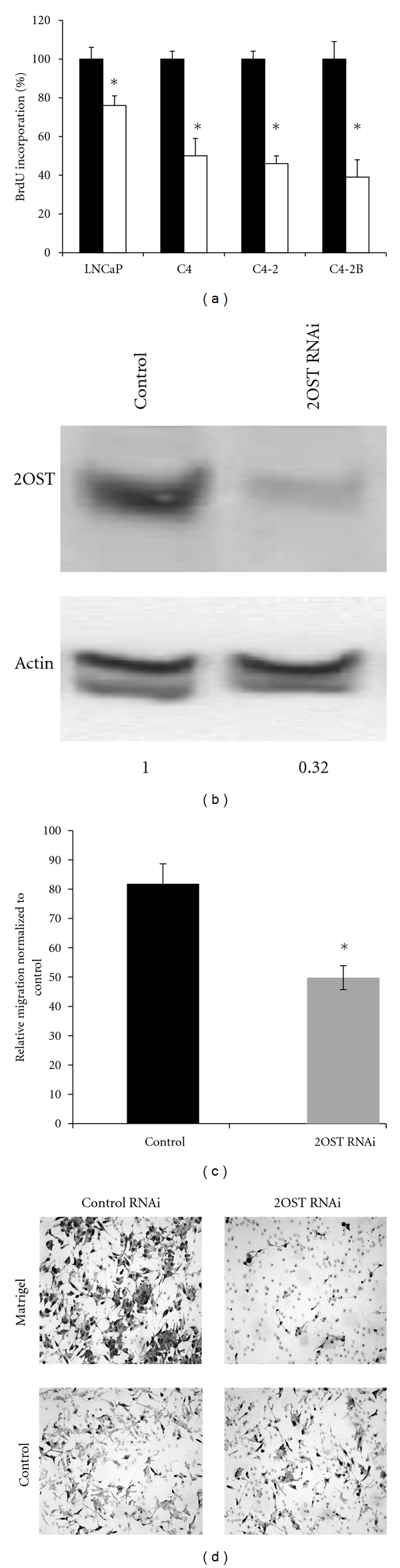
Inhibition by 2OST siRNA decreases prostate cancer cell proliferation and migration. (a) BrdU incorporation in LNCaP, C4, C4-2, and C4-2B cell lines. The cell lines are listed in order of increasing metastatic potential from left to right on the graph. All samples were normalized to controls (scrambled siRNA treated). Black bars represent control samples transfected with scrambled siRNA. White bars represent samples transfected with 2OST siRNA. Error bars represent *n* = 3 independent samples and standard deviation. Asterisk indicates *P* < 0.05. (b) Western blot for verification of 2OST siRNA. Levels of 2OST in each sample were normalized to actin by densitometry. Values are representative of two independent experiments. (c) Inhibition by 2OST siRNA decreases Matrigel invasion by C4-2B cells. The number of C4-2B cells that migrated through Matrigel was counted for control cells (scrambled siRNA treated) and experimental cells (2OST siRNA treated) in four separate fields in three independent experiments. The same experiment was performed with control inserts. The average number of invading cells (*n* = 12) was normalized to the average number of cells on control inserts (*n* = 12) to determine percent invasion. Average % invasion for control and 2OST siRNA cells were 81.8 ± 6.88 and 49.8 ± 4.08, respectively (*P* < 0.01, asterisk). (d) Representative images of C4-2B cells used in Matrigel assay. Cells that migrated through the matrigel in either control or 2OST siRNA samples are shown in (a,b).Cells used in control inserts are shown on (c,d).

The correlation between 2OST expression and metastatic potential suggested that 2OST might play a role in metastasis-associated processes of the LNCaP-C4-2B cell line series. To investigate this possibility, we asked if changes in the level of 2OST would affect cellular proliferation. We assayed cell proliferation using BrdU incorporation in each cell line either transfected with a control siRNA (black bars) or siRNA directed against 2OST (white bars) (Figure 2(a)). We found that proliferation decreased in each of the four cell lines as a result of the 2OST siRNA. The proliferation of the most advanced cell lines was the most sensitive to knockdown of 2OST expression. To verify a decrease in the amount of 2OST protein as a result of the siRNA treatment, Western blot analysis was performed on C4-2B cells treated with either the control siRNA or 2OST siRNA. We were able to reproducibly detect an approximate decrease of 50% in levels of 2OST protein (Figure 2(b)). In summary, the 2OST enzyme is necessary for optimal proliferation of prostate cancer cells in the LNCaP-C4-2B cell line series.

Along with proliferation, cell invasion is one of the principal processes that are required for cancer progression and metastasis. The correlation we observed between 2OST expression and metastatic potential suggested that a decrease in 2OST expression would lead to a decrease in the invasive potential of these cells. To determine if inhibition of 2OST by siRNA affects prostate cancer cell invasion, we evaluated the invasive potential of the most metastatic cell line C4-2B in an *in vitro* Matrigel invasion assay. C4-2B was chosen for this experiment due to its proliferation being the most sensitive to 2OST siRNA and it having the highest levels of 2OST expression. Approximately 80% of C4-2B cells treated with control siRNA invaded through Matrigel demonstrating its high invasive potential (Figure 2(c)). Inhibition of 2OST by siRNA in C4-2B cells resulted in a significant decrease in the mean number of cells that invaded through the Matrigel (*P* < 0.01). Percent invasion of 2OST siRNA treated cells dropped to approximately 50% (Figure 2(c)). Figure 2(d) shows a representative field of C4-2B cells treated with either control siRNA (a,c)or the 2OST siRNA(b,d). This data demonstrates that optimal *in vitro* migration of highly metastatic prostate cancer cells requires 2OST.

### 3.4. Increased Actin and E-cadherin Accumulation in C4-2B Cells Treated with 2OST siRNA

**Figure 3:**
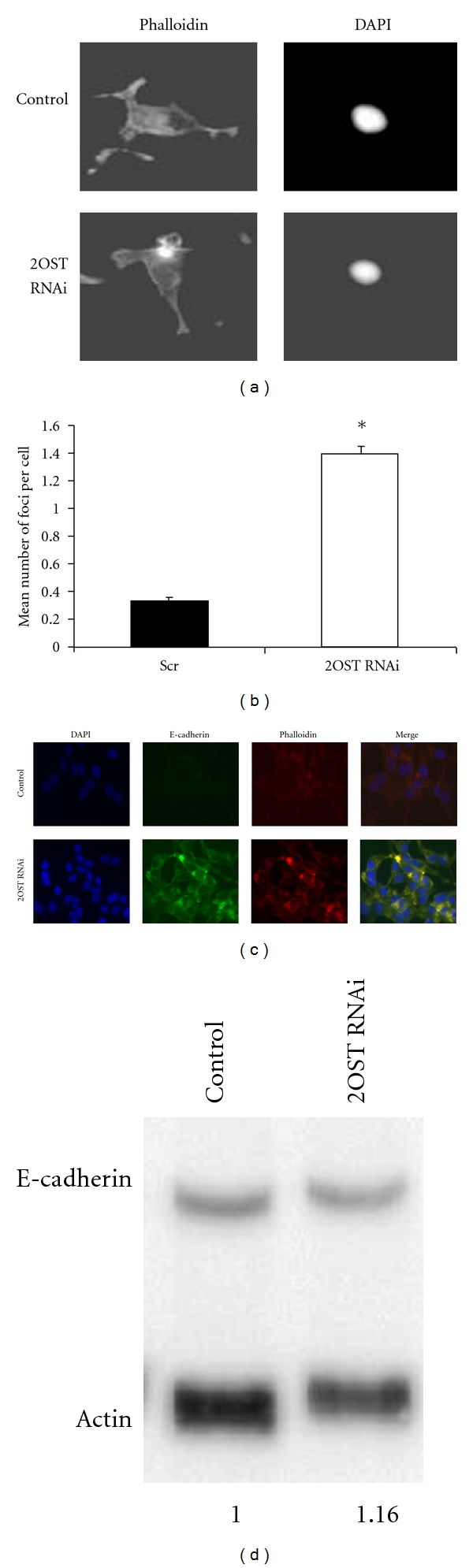
Inhibition by 2OST siRNA increases actin foci in C4-2B cells. (a) Fluorescence staining for F-actin (left panels) and nuclei (right panels) in C4-2B cells treated with 2OST siRNA (bottom row) or control siRNA (top row). Notice the clustering of F-actin into foci in the 2OST siRNA treated cells. (b) Quantitation of number of actin foci present per cell in C4-2B cells treated with scrambled siRNA (control, black bar) and 2OST siRNA (white bar). Mean number of foci per cell ± sem are 0.33 ± 0.03 and 1.14 ± 0.06 respectively (*n* = 15). Asterisk indicates *P* < 0.05. (c) Inhibition by 2OST siRNA increases E-cadherin staining that colocalizes with actin foci in C4-2B cells. Fluorescence immunohistochemistry for DAPI stained nuclei (left column); E-cadherin (second column), F-actin (third column), and merged images (fourth column) in C4-2B cells treated with 2OST siRNA (bottom row) or scrambled siRNA (top row). Notice accumulation of E-cadherin between cells treated with 2OST siRNA. (d) Inhibition by 2OST siRNA does not result in significant upregulation of E-cadherin protein. Western blot of E-cadherin in samples treated with scrambled siRNA or 2OST siRNA. Levels of E-cadherin were normalized to actin. Values represent two independent experiments.

Cell adhesion in epithelial cells is provided in part by the formation of adherens junctions. These cell to cell contacts consist of the proteins E-cadherin, *β*-catenin, *α*-catenin, and actin filaments [26]. Adherens junctions are formed between epithelial cells, such as those in the prostatic duct, when plasma-membrane spanning E-cadherin proteins recruit catenin molecules, which in turn bind to actin filaments. The junctions are stabilized by the formation of E-cadherin clusters and then further stabilized by the accumulation of actin filaments at the contact region [27, 28]. During the progression of metastatic disease, epithelial cells lose cell-cell contacts via adherens junctions and become fibroblast like in a process called the epithelial-mesenchymal transformation (EMT) [27]. Due to the decreased invasion of C4-2B cells that have been treated with 2OST siRNA, we hypothesized that 2OST acts to repress adherens junction formation. To test this hypothesis, we evaluated the accumulation of actin via phalloidin staining in C4-2B cells treated either with control siRNA or 2OST siRNA (Figures 3(a)-3(b)). A significant increase in the mean number of actin foci per cell in C4-2B cells treated with 2OST siRNA was observed (Figure 3(b)).Figure 3(a) shows representative cells of each treatment. Notice the accumulation of phalloidin staining in the 2OST siRNA treated cell.

To determine if these actin foci might correspond to the formation of adherens junctions, we evaluated actin-E-cadherin double labeling in C4-2B cells treated with either control siRNA or 2OST siRNA (Figure 3(c)). Control siRNA treated cells had very low levels of both actin and E-cadherin accumulation (a,b).2OST siRNA treated cells once again had accumulation of actin as shown by phalloidin staining (bottom row, third panel from left). These cells also had significantly increased levels of E-cadherin accumulation at the membrane, especially in the same regions that had increased actin accumulation (bottom panels, second and fourth panes from left). These results suggest that the inhibition of 2OST by siRNA is facilitating the formation of adherens junctions and thus a possible reversal of EMT. 

The possibility exists that the loss of 2OST is allowing for an increase in E-cadherin expression that in turn permits formation of these junctions. To test this possibility, we performed Western blot analysis of E-cadherin protein in C4-2B cells treated with either control siRNA or 2OST siRNA (Figure 3(d)). No significant increase in total cellular E-cadherin protein was observed in cells treated with 2OST siRNA. This result suggests that the localization of available E-cadherin in C4-2B cells changes from a more diffuse pattern to accumulation of foci when treated with 2OST siRNA.

### 3.5. Inhibition by 2OST siRNA Results in Decreased Growth Factor Signaling and Complex Formation between Perlecan and SHH in Prostate Cancer Cells

**Figure 4:**
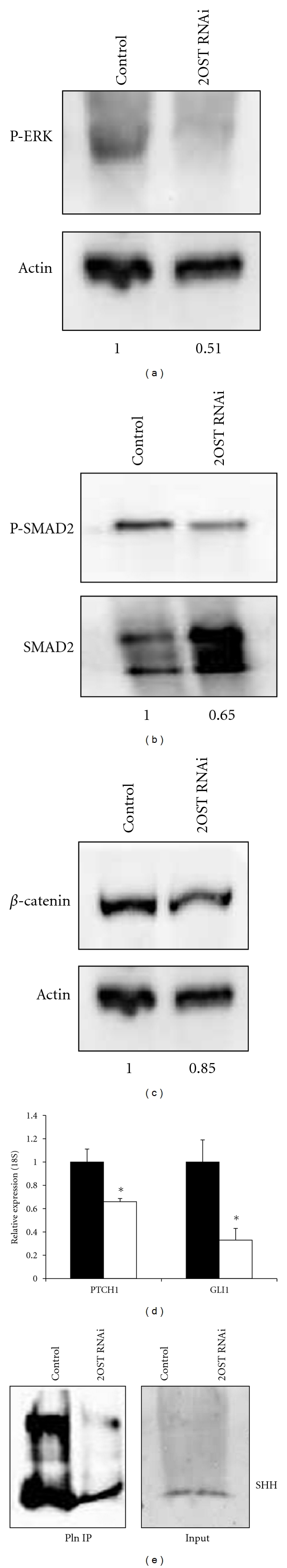
2OST modulates growth factor signaling in cell line model of prostate cancer progression. (a) Decreased FGF signaling in cells treated with 2OST siRNA. Western blotting for phospho-ERK and *β*-actin performed as described in Materials and Methods. Levels of p-ERK are normalized to *β*-actin. Densitometry figures are shown below each sample and are representative of two independent experiments. (b) Decreased TGF*β* signaling in cells treated with 2OST siRNA. Levels of p-SMAD2 normalized to total SMAD2. (c) Decreased Wnt signaling in cells treated with 2OST siRNA. Levels of *β*-catenin normalized to *β*-actin. (d) Decreased SHH signaling in cells treated with 2OST siRNA. Real-time PCR analysis of SHH pathway response genes PTCH and GLI1 was performed. Error bars indicate standard deviation. Asterisk indicates *P* < 0.05. (e) Decreased complex formation between SHH and Pln. Pln was immunoprecipitated from conditioned media from either control siRNA or 2OST siRNA cells. Left pane shows western blot for SHH bound to equal amounts of Pln. Right panel shows equal amounts of SHH in input samples from coimmunoprecipitation.

 To ask if 2OST modulates growth factor signaling in C4-2B cells, we used commonly used assays to determine levels of FGF, TGF*β*, Wnt, and Sonic Hedgehog (SHH) signaling in cells transfected with 2OST siRNA. To determine the effect of 2OST siRNA on FGF signaling, Western blots were performed analyzing levels of phospho-ERK. Densitometry reveals that phospho-ERK levels were decreased 50% in C4-2B as a result of 2OST inhibition by siRNA when using actin as normalization controls (Figure 4(a)). We then assayed the effect of 2OST knockdown on TGF*β* signaling by performing western blots for phospho-SMAD2 while using total SMAD2 as a normalization control. Densitometry analysis reveals that levels of phospho-SMAD were decreased approximately 35% in C4-2B transfected with 2OST siRNA (Figure 4(b)). We assayed levels of *β*-catenin as readout of the effect of 2OST siRNA on Wnt signaling. Levels of *β*-catenin were only decreased approximately 15% in C4-2B transfected with 2OST siRNA compared to controls (Figure 4(c)). In summary, we propose that inhibition of 2OST siRNA has significant effects on FGF and TGF*β* signaling but not on Wnt signaling.

Finally, to determine the effect of 2OST siRNA on SHH signaling, we performed qRT-PCR on RNA isolated from C4-2B cells treated with either control siRNA (black bars) or 2OST siRNA (white bars) and evaluated levels of the response genes *PTCH* and *GLI1 *(Figure 4(d)). We demonstrate that *PTCH* levels decrease approximately 40% and *GLI1* levels decrease approximately 70% as a result of 2OST knockdown. Our group has previously shown that SHH binds more readily to Pln secreted from the highly metastatic C4-2B as compared to Pln secreted from the weakly tumorigenic LNCaP cell line [8]. To determine if the 2OST enzyme is required for optimal Pln-SHH complex formation in C4-2B, we performed coimmunoprecipitations in which Pln protein was pulled down from conditioned medium of C4-2B cells treated with either control siRNA or 2OST siRNA. Western blotting to determine the levels of SHH bound to equivalent levels of Pln in each treatment reveals a significant decrease in Pln-SHH complex formation in samples treated with 2OST siRNA as compared to control siRNA (Figure 4(e)). Overall, our results suggest that the 2OST enzyme is needed for optimal growth factor signaling in the highly metastatic prostate cancer cell line C4-2B.

### 3.6. HIF1*α* Stimulates Expression of 2OST by Directly Binding Promoter in C4-2B

**Figure 5:**
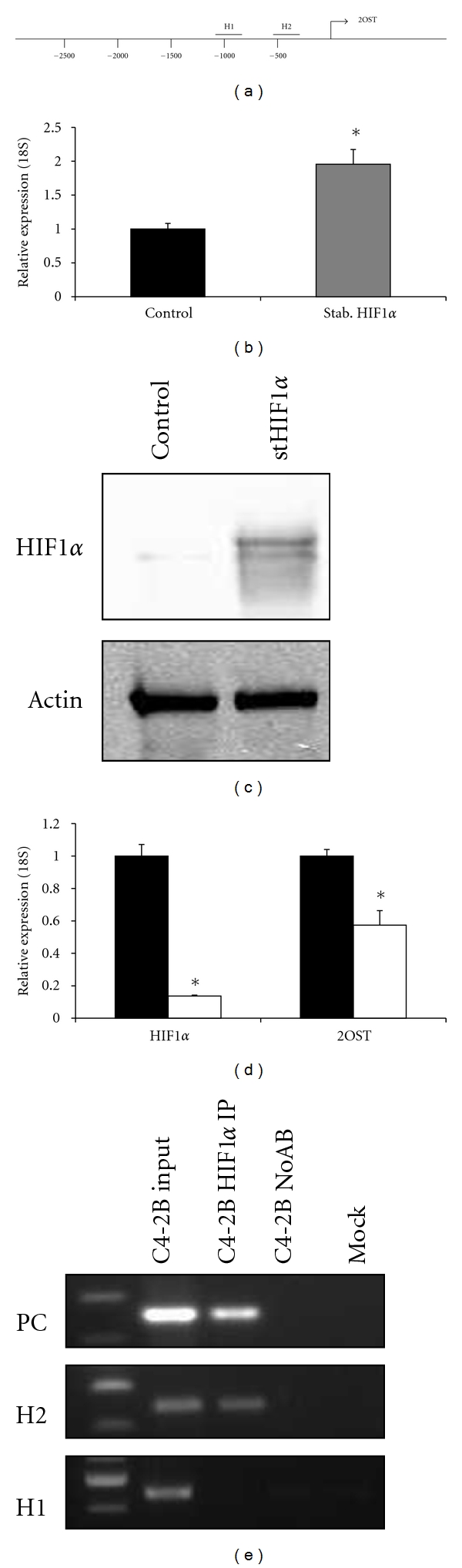
HIF1*α* activates 2OST expression in model of prostate cancer progression. (a) Schematic of 2OST promoter with regions of HREs (H1 and H2). (b) C4-2B cells were transfected with vector expressing stHIF1*α*. Real-time PCR for 2OST levels normalized to 18S levels in each sample. Black bars represent control vector alone transfected samples and gray bars represent stHIF1*α* transfected samples. Error bars indicate standard deviation. (c) Western blot for HIF1*α* shows increased accumulation of stabilized transcription factor in cells transfected with stHIF1*α* vector. (d) Inhibition of endogenous HIF1*α* expression by siRNA results in decreased levels of 2OST mRNA. Real-time PCR analysis of HIF1*α* and 2OST normalized to levels of 18S. Black bars represent samples treated with scrambled siRNA-treated samples, white bars represent levels of either HIF1*α* or 2OST in HIF1*α* siRNA-treated cells. Error bars indicate standard deviation. Asterisk indicates *P* < 0.05. (e) HIF1*α* binds directly to the 2OST promoter at predicted HREs. Chromatin Immunoprecipitation analysis of C4-2B total chromatin, HIF1*α* IP, no antibody (NoAB), and Mock samples. Samples were analyzed by PCR with primers flanking each HRE site. Positive control primers were used from the VEGF promoter [33].

The cellular stress hypoxia has been shown to correlate with increased tumor invasiveness and metastatic potential [29]. Hypoxic stress increases the stability of the transcription factor HIF1*α*, allowing it to activate gene expression; however, even prostate cancer cell lines grown under normoxic conditions have been demonstrated to have high levels of HIF1*α* protein [30, 31]. This may be in part due to the potential of reactive oxygen species to stabilize HIF1*α* as well as the ability of androgen signaling to stimulate HIF1*α* activity [21, 32]. To investigate whether the hypoxic stress-induced transcription factor HIF1*α* activates 2OST expression, we analyzed the sequence of the proximal 2OST promoter and found putative HRE sequences approximately 1000 bases (H1) and 500 bases (H2) upstream of the transcription start site (Figure 5(a)). We then asked if overexpression of HIF1*α* would be able to activate transcription of the 2OST gene. To answer this question, we evaluated levels of 2OST mRNA via qRT-PCR in C4-2B cells transfected with either an empty control vector (black bar) or a vector expressing a stabilized form of HIF1*α* (gray bar). It was found that 2OST levels increased approximately twofold in cells overexpressing the stabilized HIF1*α* (Figure 5(b)). To verify accumulation of HIF1*α* due to the overexpression vector, Western blot analysis was performed and reveals significantly increased levels of HIF1*α* as a result of the stabilized HIF1*α* transgene (Figure 5(c)). To determine if siRNA-mediated knockdown of endogenous HIF1*α* expression affected levels of 2OST mRNA, we analyzed levels of both HIF1*α* and 2OST mRNA in samples transfected with HIF1*α* siRNA (white bars) compared to control siRNA samples (black bars). HIF1*α* levels were decreased 90%, while 2OST mRNA was decreased 45% as a result of the HIF1*α* siRNA (Figure 5(d)). These results suggest that the HIF1*α* transcription factor activates expression of the 2OST gene in C4-2B prostate cancer cells. 

We then asked if the effect of HIF1*α* on 2OST transcription was direct or indirect. To answer, this chromatin immunoprecipitation (ChIP) assays were performed to evaluate physical interactions between transcription factor and promoter at the putative HREs. HIF1*α* has previously been shown to directly bind the VEGF promoter [33]. Primers for the VEGF promoter flanking HREs were used as a positive control (PC) and PCR analysis of chromatin pulled down with HIF1*α* IP demonstrated that HIF1*α* does indeed bind this promoter in C4-2B. PCR analysis using primers flanking the H1 and H2 regions of the 2OST promoter showed that HIF1*α* binds directly to the proximal H2 site but no physical interaction was detected at the H1 site (Figure 5(e)). Overall these results suggest that HIF1*α* activates 2OST transcription by directly binding its promoter.

### 3.7. p38 MAPK Signaling and ATF2 Stimulate Transcription of 2OST in C4-2B

**Figure 6:**
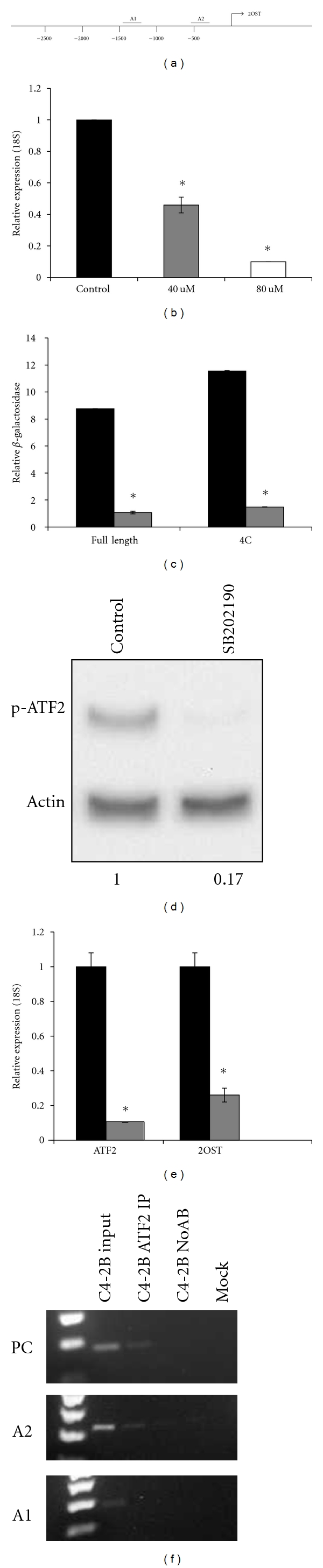
Inhibition of p38 MAPK or ATF2 results in decrease in 2OST mRNA. (a) Schematic of 2OST promoter with regions of predicted ATF2 binding sites (A1 and A2). (b) Real-time PCR analysis of 2OST levels in cells treated with either DMSO (control, black bars), 40 *μ*M (gray bars), or 80 uM SB202190 (specific p38 MAPK inhibitor, white bars) for 12 hours. Error bars indicate standard deviation. (c) 2OST promoter *β*-galactosidase reporter assay in cells treated with either DMSO control (black bars) or 80 *μ*M SB202190 (gray bars). Full-length 2OST promoter represents region 2500 bases upstream to 500 bases downstream of transcription start site. 4C represents region 1500 bases upstream to 500 bases downstream from start site. Error bars indicate standard deviation. (d) Western blot showing decreased levels of phospho-ATF2 in cells treated with 80 *μ*M SB202190. (e) Inhibition of ATF2 by siRNA results in decreased levels of 2OST mRNA. Real-time PCR analysis of ATF2 and 2OST normalized to levels of 18S. Black bars represent samples treated with scrambled siRNA, gray bars represent either ATF2 or 2OST levels in ATF2 siRNA-treated cells. Error bars indicate standard deviation. Asterisk indicates *P* < 0.05. (f) Chromatin Immunoprecipitation analysis of C4-2B total chromatin, ATF2 IP, no antibody (NoAB), and Mock samples. Samples were analyzed by PCR with primers flanking each predicted ATF2 site. Positive control primers were used from the human insulin promoter [36].

Accumulation of ROS and the resulting oxidative stress has been shown to activate p38 MAP kinase [34]. Activation of p38 MAPK is also important for the malignant phenotype in prostate cancer cells, and this is due in part to activation of the transcription factor ATF2 by phosphorylation [35]. We asked if ATF2 could be a possible transcriptional activator of 2OST expression. Our analysis of the sequence of the 2OST promoter showed two possible regions of ATF2 binding sites that have been labeled A1 and A2 (Figure 6(a)). To determine if p38 MAPK signaling was involved in 2OST transcriptional activation, we evaluated levels of 2OST mRNA by qRT-PCR in cells treated with either DMSO control (black bar) or increasing concentrations of the specific p38 inhibitor SB202190 for 12 hours. We found that treatment with 40 *μ*M SB202190 (gray bar) resulted in a 55% decrease in 2OST levels, while treatment with 80 *μ*M inhibitor (white bar) resulted in a 90% decrease (Figures 4-3(b)). This dose-dependent effect of p38 MAPK inhibitor suggests that signaling from the ROS-inducible protein kinase is important for optimal 2OST expression. To verify this effect, we produced two different *β*-galactosidase reporter constructs to assay for 2OST promoter activity. The “full-length” promoter represents the region from 2500 bases upstream of the transcription start to 435 bases downstream and the “4C” promoter represents the region from 1500 bases upstream to 435 bases downstream. p2OST promoter activity was assayed in C4-2B cells transfected with one of the reporter constructs treated with either DMSO control (black bars) or 80 *μ*M SB202190 for 12 hours (gray bars) (Figure 6(c)). We found that treatment with the p38 inhibitor resulted in a significant decrease in promoter activity with both constructs. In addition, upon comparison of results from the “full-length” reporter and the “4C” reporter it became evident that deletion of the 1000 bases most 5′ in our promoter construct leads to an increase in 2OST reporter expression, suggesting the existence of a previously unsuspected inhibitory sequence between −1500 and −2500.

We then asked if inhibition of p38 MAPK leads to a decrease in active ATF2 transcription factor in C4-2B. To answer this, Western blot analysis was performed evaluating levels of phospho-ATF2 in cells treated with DMSO control or 80 *μ*M SB202190 for 12 hours. We observed a significant decrease in phosphorylated ATF2 levels as a result of p38 inhibition (Figure 6(d)). To determine if ATF2 is acting to stimulate 2OST expression, we assayed both ATF2 and 2OST mRNA levels in cells treated with ATF2 siRNA (gray bars) or the negative control siRNA (black bars) (Figure 6(e)). The results demonstrate that while ATF2 levels were knocked down 90% 2OST levels were decreased approximately 75% by the siRNA treatment. This suggests that p38 MAPK activates ATF2, which in turn stimulates 2OST expression in the C4-2B cell line. 

We then asked if the effect of ATF2 was direct or indirect by performing ChIP assays on the 2OST promoter. Primers flanking ATF2 binding sites in the insulin promoter were used as a positive control (PC) [36]. PCR analysis with primers flanking the putative ATF2 binding sites in the 2OST promoter demonstrates a physical interaction between ATF2 and the promoter at the A2 site (Figure 6(f)).This result suggests that the effect of ATF2 on 2OST expression is direct by binding of the proximal A2 site.

### 3.8. NF*κ*B Indirectly Activates 2OST Expression in C4-2B

**Figure 7:**
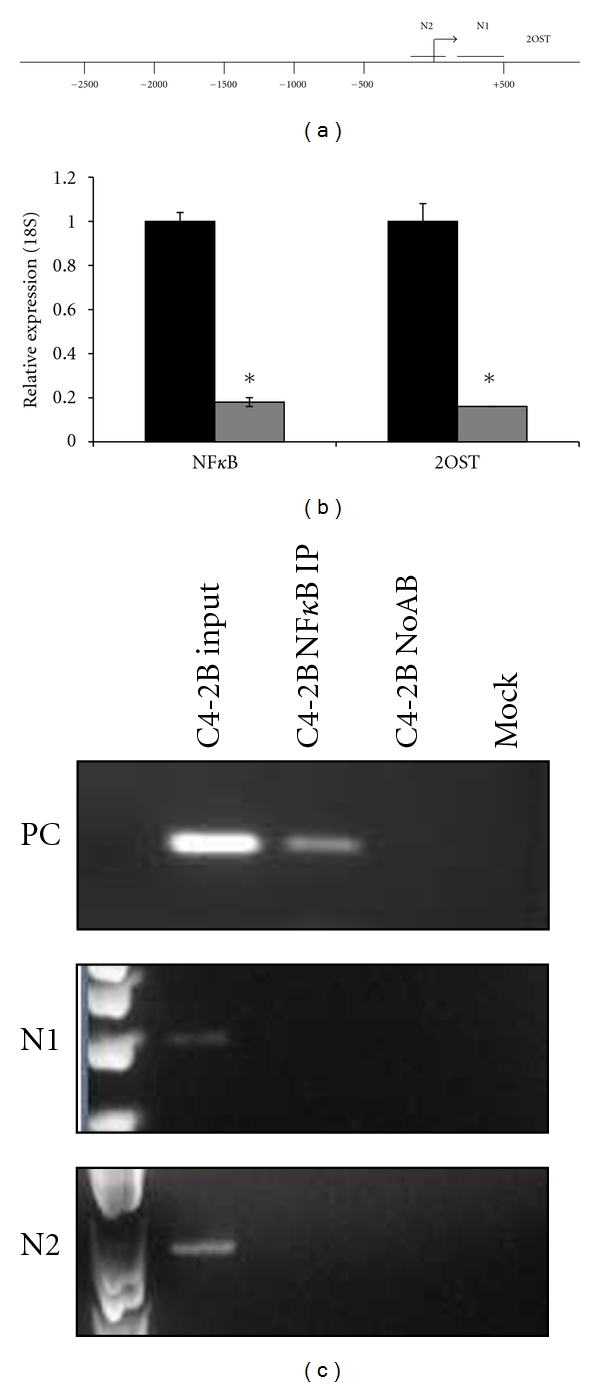
Inhibition of NF*κ*B by siRNA results in decreased 2OST mRNA. (a) Schematic representation of 2OST promoter with regions of predicted NF*κ*B-binding sites (N1 and N2). (b) Inhibition of NF*κ*B by siRNA results in decreased levels of 2OST mRNA. Real-time PCR analysis of NF*κ*B and 2OST normalized to levels of 18S. Black bars represent samples treated with scrambled siRNA treated samples and gray bars represent either NF*κ*B or 2OST levels in NF*κ*B siRNA treated cells. Error bars indicate standard deviation. Asterisk indicates *P* < 0.05.(c)NF*κ*B does not bind directly to the 2OST promoter at predicted binding sites in C4-2B. Chromatin immunoprecipitation analysis of C4-2B total chromatin, NF*κ*B IP, no antibody (NoAB), and Mock samples. Samples were analyzed by PCR with primers flanking each predicted NF*κ*B site. Positive control primers were used from the PPM1D promoter [37].

The ROS-inducible transcription factor NF*κ*B is another candidate transcription factor to induce 2OST expression. Analysis of the 2OST promoter reveals two regions that contained putative NF*κ*B binding sites (Figure 7(a), N1 and N2). To determine if knockdown of NF*κ*B expression by siRNA results in a decrease in 2OST expression, we assayed NF*κ*B and 2OST mRNA levels in cells treated with NF*κ*B siRNA (gray bars) or control siRNA (black bars) (Figure 7(b)). NF*κ*B levels were successfully knocked down ~80% by the siRNA treatment while 2OST levels also decreased ~80%. These results indicate that NF*κ*B induces 2OST expression in C4-2B prostate cancer cells.

To determine if NF*κ*B binds directly to the 2OST promoter, we performed ChIP to assay for physical interaction at the promoter (Figure 7(c)). Primers from the PPM1D promoter were used as a positive control (PC) for direct NF*κ*B binding [37]. PCR analysis with primers flanking either the N1 or N2 regions of 2OST promoter demonstrated that NF*κ*B does not bind directly to either putative binding site. These results indicate that the effect of NF*κ*B on 2OST transcription is indirect in C4-2B.

## 4. Discussion

Studies analyzing the complete loss of HS in regards to organismal and molecular phenotypes show that HS is very important for biological processes in development and disease. In an attempt to look more closely at HS fine structure, Merry et al. described the molecular phenotypes of 2OST-null mice [38]. They observed renal agenesis as well as eye and skeletal defects. Recently, it was found that 2OST is essential for FGF signaling required in chick limb bud outgrowth and development [39]. 

**Figure 8:**
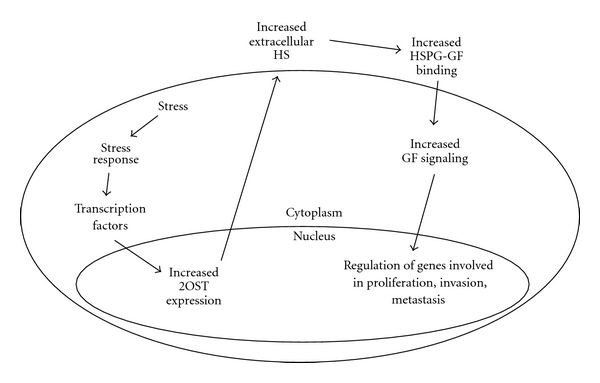
Alternative model for increased extracellular HS in prostate cancer progression.

We have chosen to analyze the effect of changes in HS fine structure via 2OST siRNA on prostate cancer cell proliferation and invasion to ask how changes in heparan sulfation may arise during cancer progression (Figure 8). Our studies have demonstrated that 2OST is required for maximal proliferation and invasion of cells in the LNCaP-C4-2B model. We also show that a knockdown of 2OST expression coincides with an increase in actin and E-cadherin accumulation at the cell surface, both markers of adherens junction formation. Our results suggest that 2OST is upregulated at the transcriptional level as cells increase in metastatic potential. We hypothesized that stress conditions such as hypoxia and ROS production, known to accompany cancer progression, and the cellular response to these stresses contribute to increased 2OST expression. We show that the stress-inducible transcription factors HIF1*α*, ATF2 and NF*κ*B are required for maximal 2OST expression. Our results demonstrate that the ROS-inducible protein kinase p38 MAPK is also required. We propose that increased 2OST expression allows for increased sulfation of HS chains on HSPGs, which allows for increased growth factor-HSPG binding. Our group previously demonstrated that binding of the SHH growth factor to Perlecan is upregulated in highly metastatic cells. In this study, we show that 2OST is required for this interaction in C4-2B cells. As a result of increased growth factor-HSPG binding, we propose that HS-dependent growth factor pathways require 2OST. We show that the SHH, FGF, and TGF*β* pathways all require 2OST for optimal signaling in highly metastatic C4-2B cells. 

Previous studies from our group indicated that 54% of malignant human prostate tumors displayed increased levels of Perlecan, which contributed to increased proliferation and SHH signaling [8]. In the current study, we characterize a cell line model of prostate cancer progression that shows no increase in Perlecan expression in an attempt to model the 46% of tumors that display basal levels of Perlecan. We introduce a plausible alternative mechanism to achieve metastasis through upregulation of 2OST. Another possibility would be to increase the expression of other HSPGs such as Glypican and Syndecan. To date no correlation between Glypican and prostate cancer progression has been published. Two recent studies suggest that Syndecan-1 correlates with increasing metastatic potential in prostate cancer patients [6, 40]. Syndecan-1 may also be a target of 2OST function, thereby contributing to prostate cancer progression. 

2OST is unique among the HS sulfation enzymes in humans due to its encoding by a single gene rather than a gene family. This relative simplicity will facilitate its use as both a marker and predictor of prostate cancer progression in human biopsies. Furthermore, the fact that knockdown of 2OST expression in the most malignant cell line, C4-2B, caused those cells to become dramatically less invasive and proliferative make 2OST an intriguing target of therapeutic potential.

## Figures and Tables

**Table 1:** 2OST is overexpressed in prostate carcinoma compared to normal tissue. The Oncomine concept “Prostate Carcinoma versus Normal—Top 10% Overexpressed” was queried in Oncomine (https://www.oncomine.org/). 2OST (HS2ST1) was significantly overexpressed in three different expression studies.

Prostate Carcinoma versus Normal—Top 10% Overexpressed		
Study	Fold change	*P* value
Varambally prostate	3.43	0.015
Luo prostate 2	1.8	0.003
Liu prostate	1.413	0.002
